# Circadian regulator BMAL1::CLOCK promotes cell proliferation in hepatocellular carcinoma by controlling apoptosis and cell cycle

**DOI:** 10.1073/pnas.2214829120

**Published:** 2023-01-03

**Authors:** Meng Qu, Guoxin Zhang, Han Qu, Alexander Vu, Raymond Wu, Hidekazu Tsukamoto, Zhenyu Jia, Wendong Huang, Heinz-Josef Lenz, Jeremy N. Rich, Steve A. Kay

**Affiliations:** ^a^International Institutes of Medicine, The Fourth Affiliated Hospital of Zhejiang University School of Medicine, Yiwu, Zhejiang 322000, China; ^b^Department of Neurology, Keck School of Medicine, University of Southern California, Los Angeles, CA 90089; ^c^Hillman Cancer Center and Department of Neurology, University of Pittsburgh Medical Center, Pittsburgh, PA 15232; ^d^Department of Neurology, University of Pittsburgh, Pittsburgh, PA 15232; ^e^Department of Botany and Plant Sciences, University of California, Riverside, CA 92521; ^f^Southern California Research Center for Alcoholic Liver and Pancreatic Diseases and Cirrhosis and Department of Pathology, Keck School of Medicine, University of Southern California, Los Angeles, CA 90033; ^g^Graduate Program in Genetics, Genomics, and Bioinformatics, University of California, Riverside, CA 92521; ^h^Department of Diabetes Complications and Metabolism, Arthur Riggs Diabetes and Metabolism Research Institute, Beckman Research Institute, City of Hope National Medical Center, Duarte, CA 91010; ^i^Division of Oncology, Norris Comprehensive Cancer Center, University of Southern California, Los Angeles, CA 90032

**Keywords:** circadian clock, hepatocellular carcinoma, cell cycle, apoptosis

## Abstract

Hepatocellular carcinoma (HCC) remains a global health challenge whose incidence is growing worldwide. Previous evidence strongly supported the notion that the circadian clock controls physiological homeostasis of the liver and plays a key role in hepatocarcinogenesis. Despite the progress, cellular and molecular mechanisms underpinning this HCC-clock crosstalk remain unknown. Addressing this knowledge gap, we show here that although the human HCC cells Hep3B, HepG2, and Huh7 displayed variations in circadian rhythm profiles, all cells relied on the master circadian clock transcription factors, BMAL1 and CLOCK, for sustained cell growth. Down-regulating *Bmal1* or *Clock* in the HCC cells induced apoptosis and arrested cell cycle at the G_2_/M phase. Mechanistically, we found that inhibiting *Bmal1*/*Clock* induced dysregulation of the cell cycle regulators *Wee1* and *p21* which cooperatively contribute to tumor cell death. *Bmal1*/*Clock* knockdown caused downregulation of *Wee1* that led to apoptosis activation and upregulation of *p21* which arrested the cell cycle at the G_2_/M phase. Collectively, our results suggest that the circadian clock regulators BMAL1 and CLOCK promote HCC cell proliferation by controlling *Wee1* and *p21* levels, thereby preventing apoptosis and cell cycle arrest. Our findings shed light on cellular impact of the clock proteins for maintaining HCC oncogenesis and provide proof-of-principle for developing cancer therapy based on modulation of the circadian clock.

## Significance

The circadian clock modulates the expression of many protein-coding genes in most cell types, thereby playing a key role in human health. Recent discoveries have unveiled the circadian clock as a novel pathway for therapeutic intervention in cancer. Liver cancer remains a major public health concern globally and is closely associated with dysregulated circadian parameters. However, the underlying mechanism is currently unknown. Our findings establish anti-apoptotic roles of the master clock regulators BMAL1 and CLOCK in promoting proliferation of liver cancer cells and reveal an underpinning mechanism being mediated by the cancer-state essential gene *Wee1*.

Hepatocellular carcinoma (HCC), the most common form of liver cancer, is the third leading cause of cancer-related deaths worldwide. As the incidence of viral hepatitis-related liver cancer has declined due to preventive interventions and treatments, other etiologies such as non-alcoholic fatty liver disease (NAFLD) and alcohol-associated liver disease (ALD) are emerging as leading risk factors associated with rising HCC cases (1). Molecularly targeted therapeutics have demonstrated efficacy for HCC treatment; the combination of atezolizumab (anti-PD-L1 antibody) and bevacizumab (anti-vascular endothelial growth factor antibody) has become the new standard first-line treatment demonstrating superior survival and response rate compared to the prior standard of sorafenib (2). While these agents have the potential to improve patient outcomes, the efficacy is far from adequate, and the objective response rates are still low (about 25%) (3). Defining the underlying molecular mechanisms driving HCC initiation and growth is urgently needed to lay the foundation for novel molecularly targeted therapeutic approaches.

The circadian clock represents an evolutionarily conserved mechanism that achieves the coordination of physiological processes and behavior with the day-night cycles. The molecular clock centered on the transcription factor BMAL1 and its partner CLOCK (BMAL1::CLOCK) establishes rhythmic expression of most protein-coding genes in mammalian genomes, thereby gating key physiological outputs, including sleep, feeding, hormone secretion, metabolism, and immune responses (4, 5). Several studies have demonstrated that core clock genes, including *Bmal1* and *Clock*, are commonly dysregulated or mutated in cancer cells (6, 7). Epidemiological and genetic studies have uncovered reciprocal regulation of disrupted circadian homeostasis and oncogenic processes in the context of several cancer types (8–14). Importantly, modulation of the core clock by small molecules has recently emerged as a promising new approach in cancer therapy, thus providing a possible path toward experimental therapeutics (10, 14).

Due to the distinct chromatin landscape shaped by tissue-specific pioneer factors, the liver represents a physiological hub for circadian regulation (4, 15, 16). The hepatic circadian transcripts are involved in principal functions of the liver, including glucose homeostasis, lipogenesis, bile acid synthesis, mitochondrial biogenesis, oxidative metabolism, amino acid turnover, and xenobiotic detoxification (17). Chronic jet lag disturbing the circadian rhythms accelerated diethylnitrosamine (DEN)-induced liver carcinogenesis (18) and caused spontaneous HCC development following increased susceptibility to NAFLD (19). Alterations of the circadian clock genes are correlated with survival and clinical outcome of HCC patients (6). Accordingly, the circadian control of liver physiological homeostasis plays a key role in hepatocarcinogenesis. Despite the genetic correlations, the molecular functions of the clock genes implicated in HCC pathogenesis are still enigmatic, which hampers the efforts to leverage therapeutic strategies targeting clock proteins in HCC.

In the present study, we report that the core clock genes *Bmal1* and *Clock* play a pro-proliferative role in HCC by controlling cell cycle regulators. Down-regulating *Bmal1* and *Clock* induced HCC cytotoxicity, activated apoptosis, and altered cell cycle progression. *Wee1* encoding an essential kinase in the G_2_/M checkpoint and *p21^Cdkn1a/Waf1/Cip1^* (referred to as *p21* hereafter) encoding a cyclin-dependent kinase inhibitor that negatively regulate cell cycle progression are transcriptional targets of the circadian clock circuit that contribute to *Bmal1/Clock*-promoted HCC cell proliferation. *Bmal1/Clock* knockdown induced downregulation of *Wee1* leading to enhanced apoptosis. However, the G_2_/M phase arrest caused by *Bmal1/Clock* knockdown was independent of *Wee1* but associated with altered expression of the *p21* gene. Therefore, *Bmal1* and *Clock* control HCC cell proliferation through divergent molecular mechanisms that involve the inhibition of apoptosis and cell cycle arrest.

## Results

### The Core Clock Genes *Bmal1* and *Clock* Are Indispensable for HCC Cell Growth.

**Fig. 1.**
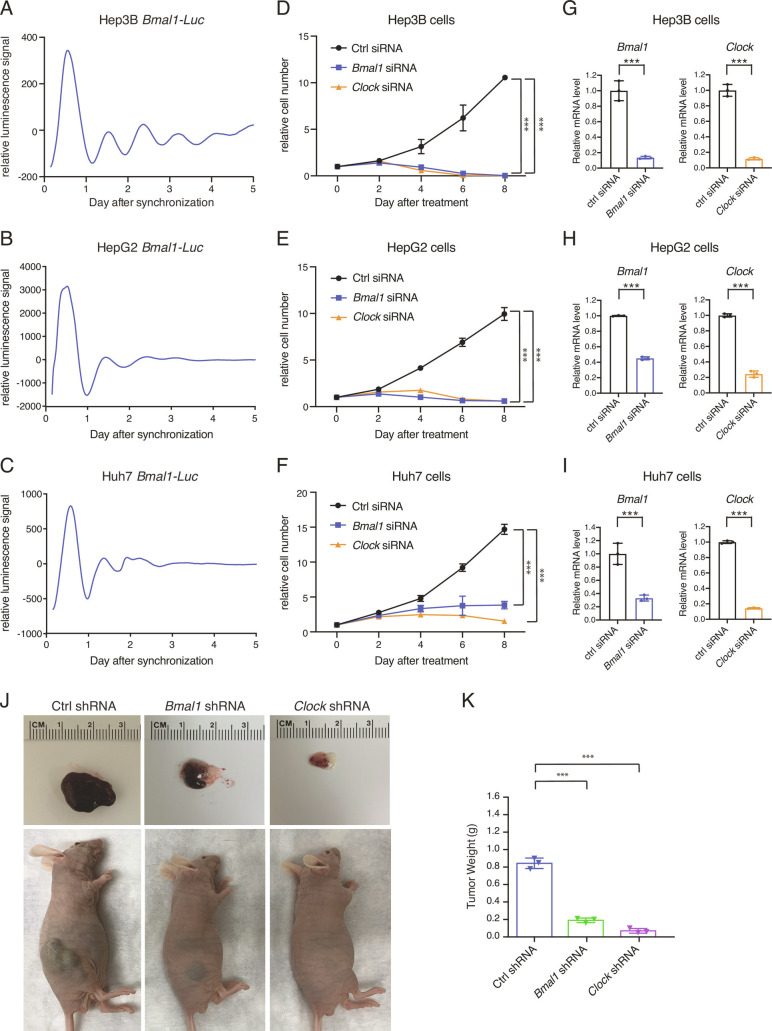
The core clock genes *Bmal1* and *Clock* are required for HCC cell growth independent of circadian oscillation. (*A*–*C*) Representative bioluminescence of *Bmal1-Luc* reporter in Hep3B (*A*), HepG2 (*B*), and Huh7 (*C*) cells synchronized by 150 nM dexamethasone (n = 3). (*D*–*F*) Relative cell numbers of Hep3B (*D*), HepG2 (*E*), and Huh7 (*F*) transfected with scramble, *Bmal1*, or *Clock* siRNA. Data are represented as mean ± SD (n = 4). Statistical significance was determined by two-way ANOVA with Tukey multiple comparison test (***P < 0.001). (*G*–*I*) Transcript level of genes in Hep3B (*G*), HepG2 (*H*), and Huh7 (*I*) transfected with scramble, *Bmal1*, or *Clock* siRNA was determined by RT-qPCR. Displayed are the means ± SD (n = 3 cell culture wells) normalized to *Rplp0* expression levels. Statistical significance was determined by a two-tailed Student’s *t* test (***P < 0.001). (*J* and *K*) NU/J nude mice were subcutaneously injected with 3 × 10^6^ Hep3B cells transduced with indicated shRNAs. Xenograft tumors were imaged (*J*) and weighed (*K*). Displayed are the means ± SD (n = 3). Statistical significance was determined by Student’s *t* test (***P < 0.001).

We first interrogated the circadian rhythms in three widely used human HCC cells, Hep3B, HepG2, and Huh7, finding each displayed a distinct pattern (Fig. 1 *A*–*C*), with the Hep3B cells oscillating most robustly and the Huh7 cells not technically cycling according to circadian parameters (*SI Appendix*, Fig. S1). To study the functional roles of the core circadian clock in the HCC cells, we targeted *Bmal1* and *Clock* by introducing siRNA-mediated gene knockdown. Despite the heterogeneous intrinsic circadian oscillations, targeting either *Bmal1* or *Clock* potently impaired the proliferation of all three HCC cell lines (Fig. 1 *D*–*I*). We validated the clock machinery dependency in vivo with Hep3B xenografts by showing that shRNA-mediated *Bmal1/Clock* knockdown strongly inhibited tumor growth (Fig. 1 *J* and *K*). Therefore, the master circadian clock transcription factor heterodimer BMAL1::CLOCK plays a critical role in HCC cell proliferation, irrespective of the robustness of circadian oscillations or other genetic backgrounds, and is relevant to impaired tumor growth in vivo.

### Targeting *Bmal1* and *Clock* Induces Apoptosis and Cell Cycle Arrest in HCC.

**Fig. 2.**
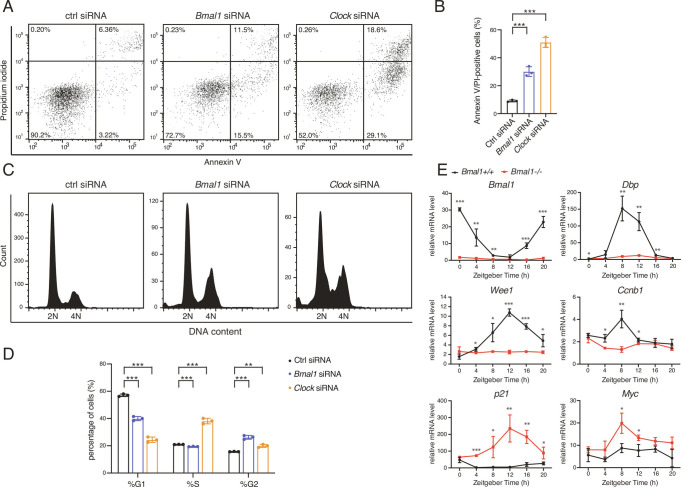
Down-regulating *Bmal1*/*Clock* activates apoptosis and G_2_/M phase cell cycle arrest. (*A*) Representative flow cytometry analysis of FITC-Annexin V/PI staining in Hep3B cells transfected with scramble, *Bmal1*, or *Clock* siRNA. (*B*) Quantification of FITC-Annexin V/PI-positive cells presented in (*A*). Displayed are the means ± SD (n = 3). Statistical significance was determined by Student’s t test (***P < 0.001). (*C*) Representative cell cycle analysis of Hep3B cells following transfection with scramble, *Bmal1*, or *Clock* siRNA. (*D*) Quantification of cell cycle phases presented in (*C*). Displayed are the means ± SD (n = 3). Statistical significance was determined by Student’s t test (**P < 0.01, ***P < 0.001). (*E*) Control and *Bmal1* knockout mouse livers were harvested at 4-h intervals throughout 24 h. Transcript level of genes was analyzed by using RT-qPCR. Displayed are the means ± SD (n = 3 or 4) normalized to non-oscillating *Rplp0* expression levels. *P*-values determined by two-tailed Student’s *t* test were displayed (*P < 0.05, **P < 0.01, ***P < 0.001).

To elucidate the molecular mechanisms underlying BMAL1::CLOCK-regulated HCC proliferation, we determined cellular effects of *Bmal1* and *Clock* knockdown. Flow-cytometric measurement of Annexin V and propidium iodide (PI) staining as well as the cleavage of CASPASE 3 revealed the activation of bonafide apoptosis upon *Bmal1* or *Clock* knockdown (Fig. 2 *A* and *B* and *SI Appendix*, Fig. S2). *Bmal1/Clock*-targeted HCC cells displayed a decreased G_1_ fraction and a concomitant increase in the G_2_/M fraction relative to cells transduced with a non-targeting control siRNA (Fig. 2 *C* and *D*), indicating inhibited cell cycle progression. To determine if similar regulation occurs in normal tissues, we performed RT-qPCR characterization of the *Bmal1* knockout mouse liver, which identified extensively disrupted transcription of cell cycle genes compared to the wild-type liver; specifically*, Wee1* and *Ccnb1* were down-regulated and *p21* and *Myc* were up-regulated (Fig. 2*E*). Therefore, BMAL1::CLOCK may control transcription of the cell cycle regulators (20).

### The Circadian Clock Contributes to Control of Cell Cycle Progression by Transcriptional Regulation of *Ccnb1*, *p21*, and *Myc*.

**Fig. 3.**
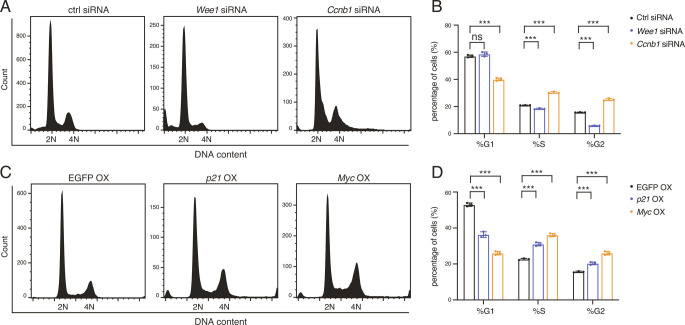
*Ccnb1* knockdown and *p21* or *Myc* overexpression cause G_2_/M phase arrest. (*A*) Representative cell cycle analysis of Hep3B cells following transfection with scramble, *Wee1*, or *Ccnb1* siRNA. (*B*) Quantification of cell cycle phases presented in (*A*). Displayed are the means ± SD (n = 3). Statistical significance was determined by Student’s *t* test (***P < 0.001; ns, not significant). (*C*) Representative cell cycle analysis of Hep3B cells following lentiviral transduction of EGFP, *p21*, or *Myc* gene. (*D*) Quantification of cell cycle phases presented in (*C*). Displayed are the means ± SD (n = 3). Statistical significance was determined by Student’s *t* test (***P < 0.001).

We were accordingly prompted to investigate whether BMAL1::CLOCK promotes the growth of HCC cells by modulating cell cycle progression and division. We first inspected the mechanism underpinning clock-regulated cell cycle progression. As cell cycle progression requires well-orchestrated transcriptional control of the cell cycle regulators (21), we characterized the impact of the altered transcription of cell cycle genes in response to *Bmal1* depletion (Fig. 2*E*). siRNA-mediated *Wee1* downregulation induced G_1_ phase accumulation (Fig. 3 *A* and *B*), which is compatible with its functional roles in activating the G_2_/M checkpoint (22). In contrast, the knockdown of *Ccnb1*, a G_2_/M-specific cyclin, reduced the fraction of cells in G_1_ phase and increased the proportion of cells in the G_2_/M phase (Fig. 3 *A* and *B*). Overexpression of *p21*, a cyclin-dependent kinase inhibitor that promotes cell cycle arrest (23), increased the fraction of cells arrested in G_2_/M phase (Fig. 3 *C* and *D*). Consistent with previous studies (24), induction of *Myc* expression increased cell population arrested in the G_2_/M phase (Fig. 3 *C* and *D*). Therefore, the G_2_/M phase cell cycle arrest resulting from *Bmal1/Clock* knockdown (Fig. 2 *C* and *D*) is likely associated with transcriptional downregulation of *Ccnb1* and upregulation of *p21* and *Myc.*

### BMAL1::CLOCK Promotes HCC Cell Proliferation by Suppressing the Cell Cycle Regulator *p21*.

**Fig. 4.**
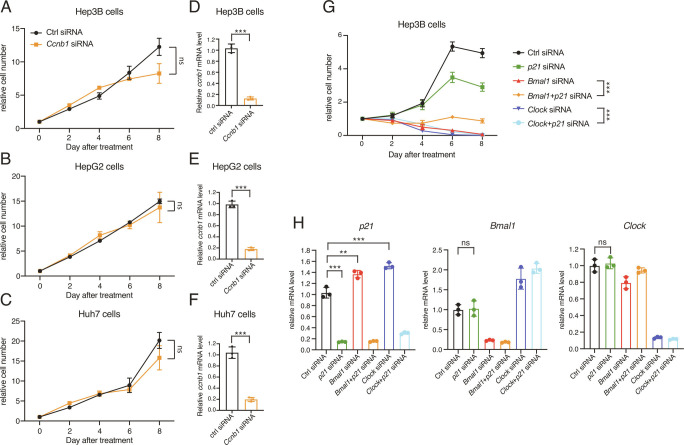
*Bmal1*/*Clock* controls HCC cell growth by suppressing *p21*. (*A*–*C*) Relative cell numbers of Hep3B (*A*), HepG2 (*B*), and Huh7 (*C*) transfected with scramble or *Ccnb1* siRNA. Data are represented as mean ± SD (n = 4). Statistical significance was determined by two-way ANOVA with Tukey multiple comparison test (ns, not significant). (*D*–*F*) Transcript level of genes in Hep3B (*D*), HepG2 (*E*), and Huh7 (*F*) transfected with scramble or *Ccnb1* siRNA was determined by RT-qPCR. Displayed are the means ± SD (n = 3 cell culture wells) normalized to *Rplp0* expression levels. Statistical significance was determined by a two-tailed Student’s *t* test (***P < 0.001). (*G*) Relative cell numbers of Hep3B transfected with scramble, *Bmal1*, *Clock*, or *p21* siRNA. Data are represented as mean ± SD (n = 4). Statistical significance was determined by two-way ANOVA with Tukey multiple comparison test (***P < 0.001). (*H*) Transcript level of genes in Hep3B transfected with scramble, *Bmal1*, *Clock*, or *p21* siRNA was determined by RT-qPCR. Displayed are the means ± SD (n = 3 cell culture wells) normalized to *Rplp0* expression levels. Statistical significance was determined by a two-tailed Student’s *t* test (**P < 0.01, ***P < 0.001; ns, not significant).

Cell cycle checkpoints regulate the rate of cell division. Next, we assessed whether the *Ccnb1, Myc,* and *p21-*associated G_2_/M phase cell cycle arrest in response to *Bmal1/Clock* downregulation reduced cell proliferation. Knockdown of *Ccnb1* alone had a minor impact on HCC cell growth (Fig. 4 *A*–*F*). Also, upregulation of the proto-oncogene *Myc* is unlikely to lead to HCC proliferation inhibition. Therefore, the G_2_/M phase arrest does not necessarily result in reduced cell proliferation *per se*, in concordance with previous reports that depending on molecular context, either inhibition or activation of the G_2_/M checkpoint can induce cell death (25).

*Bmal1* knockout increased the basal level of *p21* and reversed its peak expression from ZT0 to ZT12 (Fig. 2*E*). Gréchez-Cassiau and co-workers reported that the cyclic expression of *p21* was controlled by the RORE-binding clock transcription factors REV-ERBs and RORs and up-regulated *p21* was responsible for the decreased hepatocyte proliferation rate in the *Bmal1* knockout mouse liver (26). We found that concurrent knockdown of *p21* partially rescued HCC growth inhibition induced by *Bmal1*/*Clock* knockdown (Fig. 4 *G* and *H*). *p21* overexpression did not substantially change the activity of apoptosis in Hep3B cells (*SI Appendix*, Fig. S3), in line with previous reports (27). Collectively, our findings indicate that BMAL1::CLOCK negatively regulates the RORE-containing cell cycle regulator *p21* to maintain proliferation rate of HCC cells.

### BMAL1::CLOCK Promotes HCC Cell Proliferation by Stimulating Expression of the Cancer-state Essential Gene *Wee1*.

**Fig. 5.**
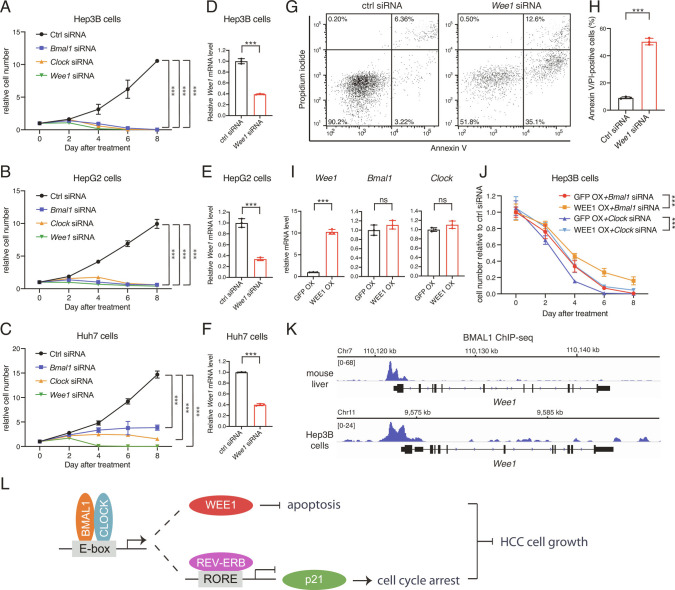
*Bmal1*/*Clock* controls HCC cell growth by activating *Wee1*. (*A*–*C*) Relative cell numbers of Hep3B (*A*), HepG2 (*B*), and Huh7 (*C*) transfected with scramble, *Bmal1*, *Clock*, or *Wee1* siRNA. Data are represented as mean ± SD (n = 4). Statistical significance was determined by two-way ANOVA with Tukey multiple comparison test (***P < 0.001). (*D*–*F*) Transcript level of genes in Hep3B (*D*), HepG2 (*E*), and Huh7 (*F*) transfected with scramble or *Wee1* siRNA were determined by RT-qPCR. Displayed are the means ± SD (n = 3 cell culture wells) normalized to *Rplp0* expression levels. Statistical significance was determined by a two-tailed Student’s *t* test (***P < 0.001). (*G*) Representative flow cytometry analysis of FITC-Annexin V/PI staining in Hep3B cells transfected with scramble or *Wee1* siRNA. (*H*) Quantification of FITC-Annexin V/PI-positive cells presented in (*G*). Displayed are the means ± SD (n = 3). Statistical significance was determined by Student’s *t* test (***P < 0.001). (*I*) Transcript level of genes in Hep3B stably overexpressing GFP or *Wee1* was determined by RT-qPCR. Displayed are the means ± SD (n = 3 cell culture wells) normalized to *Rplp0* expression levels. Statistical significance was determined by a two-tailed Student’s *t* test (***P < 0.001; ns, not significant). (*J*) Relative cell numbers of Hep3B stably overexpressing GFP or *Wee1* followed by transfection with *Bmal1* or *Clock* siRNA. Cell numbers were normalized to Hep3B cells transfected with scramble siRNA of the same time point. Data are represented as mean ± SD (n = 4). Statistical significance was determined by two-way ANOVA with Tukey multiple comparison tests (***P < 0.001). (*K*) IGV genome tracks showing BMAL1 enrichment at the *Wee1* gene loci in the mouse liver and Hep3B cells, based on normalized ChIP-seq read coverage. Track heights are indicated. (*L*) Mechanism of BMAL1::CLOCK-regulated HCC oncogenesis. BMAL1::CLOCK binds the E-box element on chromatin and activates the transcription of *Rev-erbs* and *Wee1*. REV-ERBs suppresses the expression of *p21* through binding RORE element. The inhibition of cell cycle arrest and apoptosis, which are activated by *p21* downregulation and *Wee1* upregulation, respectively, collaboratively contribute to pro-proliferative activity of BMAL1::CLOCK.

Cancer cells are reliant on WEE1-activated G_2_/M checkpoint to cope with DNA damage and avoid apoptosis (28). Analogous to *Bmal1/Clock* knockdown, we found that down-regulating *Wee1* attenuated proliferation and caused apoptosis in HCC cells (Fig. 5 *A*–*H*). As depleting *Bmal1* significantly reduced *Wee1* levels in both mouse liver and Hep3B cells (Fig. 2*E* and *SI Appendix*, Fig. S4), we asked whether *Wee1* downregulation contributed to cell lethality resulting from BMAL1::CLOCK inhibition. Overexpression of *Wee1* did not change the expression levels of *Bmal1* and *Clock* (Fig. 5*I*) but could partially rescue cell growth inhibition caused by BMAL1::CLOCK downregulation (Fig. 5*J*). *Wee1* transcription showed robust circadian oscillation in the mouse liver, peaking at the end of the day, in phase with the classical BMAL1::CLOCK target gene *Dbp* (Fig. 2*E*). BMAL1 chromatin immunoprecipitation followed by deep sequencing (ChIP-seq) indicated that BMAL1::CLOCK binds *Wee1* genes in both mouse liver and Hep3B cells (Fig. 5*K*). Therefore, *Wee1* inhibits apoptosis and contributes to BMAL1::CLOCK-controlled HCC cell growth.

## Discussion

Previous studies strongly support the crosstalk between HCC and the circadian clock. Modeling chronic jet lag to disrupt the circadian rhythms accelerated DEN-induced murine liver carcinogenesis (18) and even increased the incidence of spontaneous HCC associated with NAFLD (19). However, as chronic jet lag induced circadian desynchrony of most key clock genes, it is challenging to generalize from the results to the role of any single clock component in the observed HCC predisposition. Conversely, circadian transcript analysis of patient specimens revealed that a large body of genes cycling in noncancerous tissues, including specific core clock genes, lost oscillations in HCC tumors (29–34). Here, our results provide molecular insights into this HCC-clock crosstalk by unveiling that the core clock transcription factor BMAL1::CLOCK is hijacked by cancer cells to fuel rapid cell proliferation and inhibit apoptosis in HCC, independent of the genetic background or core clock oscillations.

Mechanistically, inhibiting *Bmal1* or *Clock* triggered systemic dysregulation of the cell cycle in HCC cells featured by activation of cell cycle arrest and apoptosis. Relative to *Bmal1*, we found knocking down *Clock* induced more apoptosis and arrested cell cycle at both the S and G_2_/M phases. It will be of interest for future studies to investigate whether the difference is because of potentially differentiated biological functions of the *Bmal1* and *Clock* genes or solely due to higher knockdown efficiency of the *Clock* siRNA (Fig. 1 *G*–*I*). Further, we provide genetic evidence supporting a scenario in which transcriptional alterations of the key cell cycle regulators *Wee1* and *p21* cooperatively contribute to cell growth inhibition in cells where BMAL1::CLOCK is down-regulated. BMAL1::CLOCK downregulation halted the cell cycle progression by up-regulating *p21* and meanwhile activated apoptosis by down-regulating *Wee1*. As BMAL1 cooperates with the hepatocyte nuclear factor HNF4A in driving the liver-specific circadian regulation of tumorigenesis (15, 16, 35, 36), BMAL1::CLOCK is a versatile player in HCC oncogenesis targeting multiple pathways. This study provides proof-of-principle for future development of novel liver cancer therapies via modulation of the circadian clock. CRYs, PERs, and REV-ERBs agonists inhibit BMAL1::CLOCK through circadian clock negative feedback loops (7). Therapeutics targeting the molecular clock has strong potential to be explored either as monotherapy or in combination with other therapies, with *Wee1* and *p21* potentially serving as molecular markers for evaluation of anticancer efficacy.

*p21*, a tumor suppressor that arrests cell growth by inhibiting cell cycle progression, is expressed at lower levels in HCC tissues than that in the corresponding normal liver (37–39). *p21* ablation induces continuous hepatocyte proliferation and enhances hepatocarcinogenesis in mice (40, 41). Thus, controlling compensatory proliferation by high levels of *p21* is likely critical to preventing HCC development. Pharmacological approaches developed for anticancer therapy targeting histone deacetylase (HDAC), PI3K-Akt, or MDM2-p53 interaction selectively induce *p21* expression, thereby leading to decreased cell viability (42). Our findings shed new light on *p21* regulation, now identifying the gene as a target of the circadian clock transcriptional network underlying the oncogenic function of BMAL1::CLOCK. Therefore, targeting *p21* for activation of cell cycle arrest presents an opportunity to potentiate HCC therapy using clock drugs as an adjuvant.

Normal cells repair damaged DNA during the G_1_/S cell cycle arrest, whereas cancer cells often have a deficient G_1_/S checkpoint and depend on a functional G_2_/M checkpoint for DNA repair and survival. The WEE1 kinase that plays a crucial role in the G_2_/M checkpoint arrest displays universally high expression in cancer. WEE1 inhibitors, including AZD1775 (MK1775), have been developed to compromise the G_2_/M checkpoint in combination with DNA damaging agents (22). In HCC tumors, WEE1 levels and kinase activity were found significantly elevated relative to surrounding cirrhotic tissues (43). Concordant with a previous study showing that BMAL1::CLOCK directly activates *Wee1* transcription in an vitro system (44), we found that BMAL1 bound the *Wee1* gene promoter (Fig. 5*K*) and *Bmal1* depletion significantly reduced the *Wee1* mRNA levels in liver cells (Fig. 2*E* and *SI Appendix*, Fig. S4). Down-regulating *Wee1* in HCC cells substantially attenuated HCC cell viability and induced apoptosis, which played an important role in the cytotoxicity effect of *Bmal1*/*Clock* inhibition (Fig. 5 *A*–*J*). Collectively, our observations support the concept that anti-apoptosis activity of the cancer-specific WEE1 contributes to BMAL1::CLOCK stimulated HCC cell proliferation. Small molecules targeting the clock are supposed to mimic WEE1 inhibitors but may provide increased specificity over the kinase modifiers for increasing DNA damage sensitivity of HCC patients.

## Materials and Methods

### Animal Experiments.

All animal care and experiments were performed under the institutional protocols approved by the Institutional Animal Care and Use Committee (IACUC) at the University of Southern California. For mouse liver RT-qPCR analysis, mice were housed in a 12-h light/12-h dark (LD) cycle with free access to food and water and age of 10 to 12 wk were used. For the xenograft transplantation model, NU/J nude mice (The Jackson Library #002019) were subcutaneously transplanted in the right flank with 3 × 10^6^ Hep3B cells transduced with a non-targeting control shRNA or shRNA targeting either *Bmal1* or *Clock*. Tumor growth was monitored for 4 wk by measuring tumor diameter and weight at the endpoint.

### Cell Culture.

Hep3B and Huh7 cells were grown in complete DMEM (Life Technologies cat. #11995065) supplemented with 10% FBS and 1% penicillin/streptomycin. HepG2 cells were grown in Ham’s F12 (Corning Cellgro 10-080-CV) supplemented with 10% FBS and 1% penicillin and streptomycin. All cells were grown in a 37 °C incubator at 5% CO_2_.

### Circadian Assays.

For circadian assays, human liver cancer cells were plated on 35-mm dishes and synchronized as previously described by a dexamethasone shock (10, 15, 16, 45). In brief, cell culture media was replaced with HEPES-buffered phenol-free DMEM media containing 100 nM dexamethasone and 100 μM D-luciferin. Dishes were covered with 40-mm glass coverslips (Fisher Scientific) and sealed with vacuum grease to prevent evaporation. Luminescence signals were monitored every 10 min using the LumiCycle luminometer (Actimetrics) at 37 °C without supplementary CO_2_. Results shown are representative of at least three independent experiments.

### Cell Viability Assay.

Cells were plated in 96-well plate at a density of ~1,000 cells per well. Relative ATP level was measured following the instruction of CellTiter-Glo Luminescent Cell Viability Assay (Promega, G7570).

### Cell Cycle Analysis and Apoptosis Assay.

Cells were collected and resuspended in Tris-buffered saline with 10 µg/mL DAPI and 0.1% nonidet P-40 detergent for flow cytometry. Cell cycle phases were analyzed using FlowJo software. Apoptosis was measured using FITC-Annexin V antibody and PI staining following manufacturer’s instruction (Life Technology, V13241). Cells were read using flow cytometry (BD Bioscience), and the results were analyzed via FlowJo.

### Quantitative RT-PCR.

Liver tissues of 10- to 12-wk-old male mice were harvested at indicated Zeitgeber times. Total RNA was isolated using TRIzol reagent according to manufacturer’s instructions (Life Technologies cat. #15596026) and then reverse transcribed to cDNA using iScript cDNA Synthesis kit (Bio-Rad cat. #1708891). We designed real-time primers spanning the exon–intron junctions using the IDT primer-designing software PrimerQuest (https://www.idtdna.com/PrimerQuest). Primer sequences are

*mRplp0:* forward GGCCCTGCACTCTCGCTTTC,
reverse TGCCAGGACGCGCTTGT;
*mBmal1*: forward CCCTAGGCCTTCATTGGATTT,
reverse GCAAAGGGCCACTGTAGTT;
*mDbp*: forward AATGACCTTTGAACCTGATCCCGCT,
reverse GCTCCAGTACTTCTCATCCTTCTGT;
*mWee1*: forward CCCACGTCGTTCGCTATTT,
reverse TCAGCTAAACTCCCACCATTAC;
*mCcnb1*: forward GACTCCCTGCTTCCTGTTATG,
reverse CTTGACAGTCATGTGCTTTGTG;
*mp21*: forward CGGAGGAACAGTCCTACTGATA,
reverse CAGGTAAGAAGTGGCAAGGAA;
*mMyc*: forward CGACTCTGAAGAAGAGCAAGAA,
reverse ATGGAGATGAGCCCGACT;
*hRplp0*: forward CGTGGAAGTGACATCGTCTT,
reverse GGATGATCTTAAGGAAGTAGTTGGA;
*hBmal1*: forward ATCCTCAACTACAGCCAGAATG,
reverse AGAGCTGCTCCTTGACTTTG;
*hClock*: forward TCTCAGACCCTTCCTCAACA,
reverse TGACCTTCTTTGCACCATCTT;
*hWee1*: forward ATTTCTCTGCGTGGGCAGAAG,
reverse CAAAAGGAGATCCTTCAACTCTGC;
*hCcnb1*: forward TGTGGATGCAGAAGATGGAG,
reverse TGGCTCTCATGTTTCCAGTG;
*hp21*: forward GCAGACCAGCATGACAGATTT,
reverse GGATTAGGGCTTCCTCTTGGA;
*hMyc*: forward AAAGGCCCCCAAGGTAGTTA,
reverse GCACAAGAGTTCCGTAGCTG.

RT-qPCR analyses were performed as described previously (15, 16, 45) with CFX384 Real-Time PCR Detection System (Bio-Rad).

### Western Blotting.

Frozen mouse liver tissue was homogenized in RIPA buffer containing 1x EDTA-free protease inhibitor cocktail (Roche) using Omni Tissue Homogenizer (Omni International). The total protein concentration was determined by Bio-Rad Protein Assay and then equalized to 15 g/L. 25 μg of total protein was used for the western blot assay, performed as previously described (15, 16, 45). Antibodies used in the western blots are anti-BMAL1 (Cell signaling, #14020), anti-Wee1 (Cell signaling, #4936), and anti-TUBULIN (Sigma-Aldrich, T0198).

### Quantification and Statistical Analysis.

The significance of differences between peak distance, period length, and gene expression was evaluated by unpaired Student’s *t* test (two-tailed), with significant differences at *P* < 0.05.

## Supplementary Material

Appendix 01 (PDF)

## Data Availability

All study data are included in the article and/or *SI Appendix*.
