# Supplementary material for: Circadian regulator BMAL1::CLOCK promotes cell proliferation in hepatocellular carcinoma by controlling apoptosis and cell cycle
Source: Proc Natl Acad Sci U S A. 2023 Jan 3;120(2):e2214829120. doi: 10.1073/pnas.2214829120 (PMC9926257; doi:10.1073/pnas.2214829120)
Supplement: Supplementary file 1 — Appendix 01 (PDF) [file pnas.2214829120.sapp.pdf]

Sup fig. 1

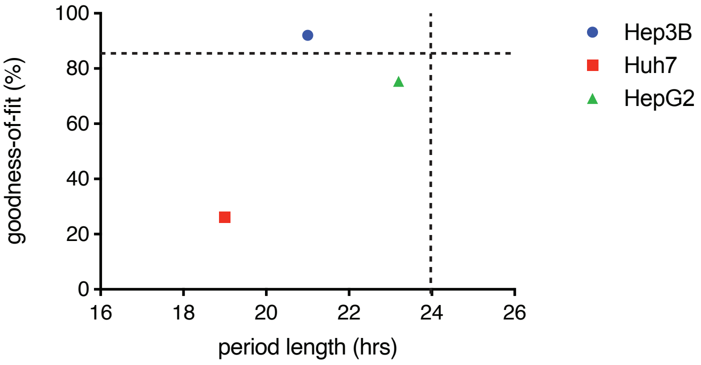

Supplementary Fig. 1. Cycle parameter analysis for Fig. 1A-C.

Sup fig. 2

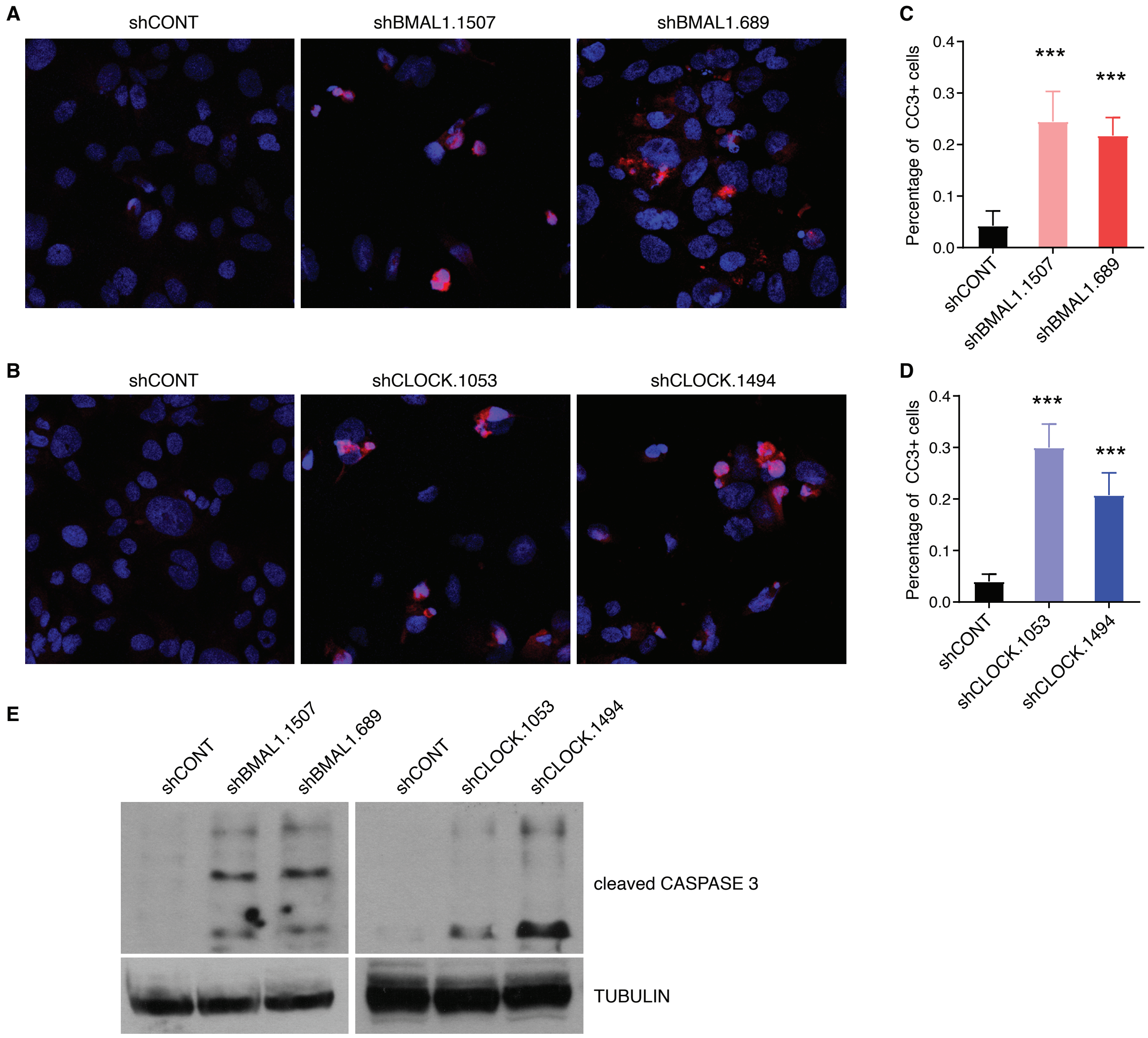

Supplementary Fig. 2. *Bmal1* or *Clock* knockdown induces apoptosis.
(A-B) Immunostaining of cleaved CASPASE 3 (red) in Hep3B transduced with shCONT, shBMAL1 **(A)** or shCLOCK **(B)**. DAPI (blue) labels the nucleus. **(C-D)** Quantification of cleaved CASPASE 3 positive cells presented in (A-B). **(E)** Immunoblot for cleaved Caspase 3 in Hep3B after *Bmal1* or *Clock* knockdown.

Sup fig. 3

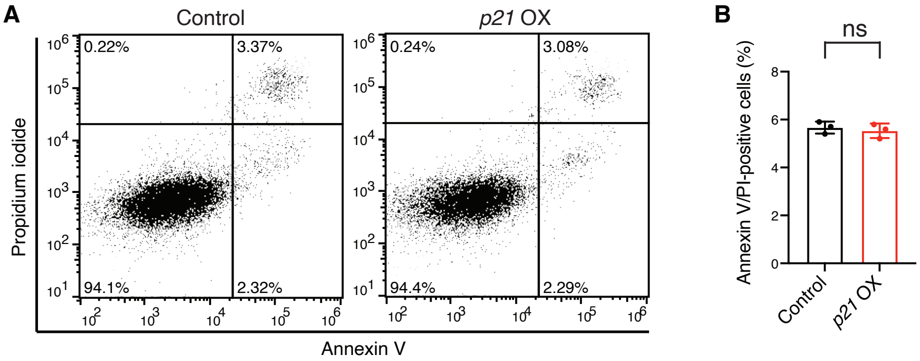

Supplementary Fig. 3. *p21* overexpression does not change apoptosis activity of Hep3B cells.

(A) Representative flow cytometry analysis of FITC-Annexin V/PI staining in Hep3B cells transduced with nothing or *p21* gene. **(B)** Quantification of FITC-Annexin V/PI-positive cells presented in **(A)**. Displayed are the means ± SD (n = 3). Statistical significance was determined by Student's t-test (ns, not significant).

Sup fig. 4

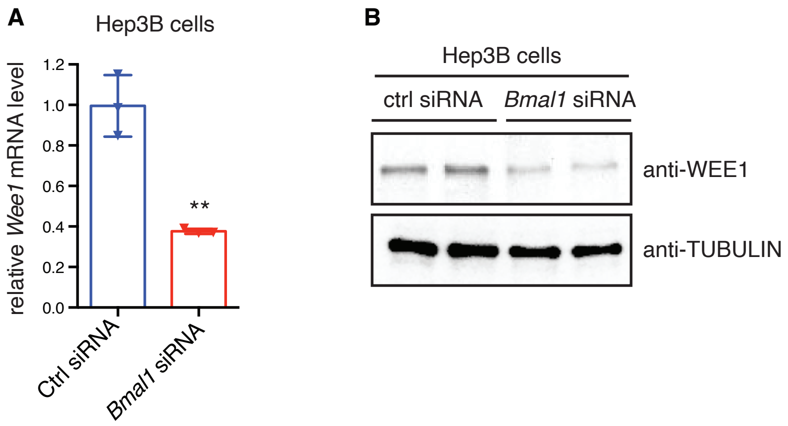

Supplementary Fig. 4. *Bmal1* knockdown leads to reduced *Wee1* expression. **(A)** Transcript level of *Wee1* in Hep3B transfected with scramble or *Bmal1* siRNA was determined by RT-qPCR. Displayed are the means ± SD (n = 3 cell culture wells) normalized to *Rplp0* expression levels. Statistical significance was determined by a two-tailed Student's t-test (**$P < 0.01$). **(B)** Western blot determining WEE1 protein level upon *Bmal1* knockdown.
